# Nonlinear gene cluster analysis with labeling for microarray gene expression data in organ development

**DOI:** 10.1186/1753-6561-5-S2-S3

**Published:** 2011-05-28

**Authors:** Martin Ehler, Vinodh N Rajapakse, Barry R Zeeberg, Brian P Brooks, Jacob Brown, Wojciech Czaja, Robert F Bonner

**Affiliations:** 1 National Institutes of Health, Eunice Kennedy Shriver National Institute of Child Health and Human Development, Section on Medical Biophysics, Bethesda MD 20892, USA; 2 University of Maryland, Department of Mathematics, Norbert Wiener Center, College Park MD 20742, USA; 3 National Institutes of Health, National Cancer Institute, Laboratory of Molecular Pharmacology, Genomics & Bioinformatics Group, Bethesda MD 20892, USA; 4 National Institutes of Health, National Eye Institute, Ophthalmic Genetics and Visual Function Branch, Bethesda MD 20892, USA

## Abstract

**Background:**

The gene networks underlying closure of the optic fissure during vertebrate eye development are not well-understood. We use a novel clustering method based on nonlinear dimension reduction with data labeling to analyze microarray data from laser capture microdissected (LCM) cells at the site and developmental stages (days 10.5 to 12.5) of optic fissure closure.

**Results:**

Our nonlinear methods created clusters of genes that mapped onto more specific biological processes and functions related to eye development as defined by Gene Ontology at lower false discovery rates than conventional linear cluster algorithms. Our new methods build on the advantages of LCM to isolate pure phenotypic populations within complex tissues in order to identify systems biology relationships among critical gene products expressed at lower copy number.

**Conclusions:**

The combination of LCM of embryonic organs, gene expression microarrays, and nonlinear dimension reduction with labeling is a potentially useful approach to extract subtle spatial and temporal co-variations within the gene regulatory networks that specify mammalian organogenesis and organ function. Our results motivate further analysis of nonlinear dimension reduction with labeling within other microarray data sets from LCM dissected tissues or other cell specific samples to determine the more general utility of our method for uncovering more specific biological functional relationships.

## Background

Common variations in genetic and epigenetic patterns among humans are associated with variations in risk for developing all common chronic diseases, a few of which have been identified from genome-wide polymorphism screens [1,2]. The functional biological robustness or its failure in disease is most likely not just reflected in a few dominant components, but in many complex interactions within gene regulatory networks. Due to the overwhelming complexity, the deeper understanding of such networks remains a major challenge in modern systems biology, a field that aims to discover and iteratively refine mechanistic models of biological processes. Biological knowledge is typically encoded in the structure and parameterization of these models. The Gene Ontology project [3,4] can help to incorporate the known biological details of gene functions into such analysis. The challenge is to reasonably approximate attributes in such models using experimental data that is complex, noisy, and often incomplete. For the purpose of acquiring biologically rich data sets, laser capture microdissection (LCM) has proven a powerful tool to isolate pure cell populations from complex heterogeneous tissue specimens [5-7]. In combination with microarray technologies, that allow the simultaneous measurement of expression levels for thousands of genes, LCM enables identifying critical gene products even if expressed at low copy numbers. Our work aims to facilitate efforts in systems biology by organizing data in ways that can suppress noise and better reveal latent, biologically meaningful structure. Coloboma is a not uncommon congenital defect of human ocular development resulting in large retinal holes which often significantly affect vision. The present paper focuses on refinements in the analysis of a temporal series of microarray data obtained from microdissected sites of retinal fissure closure in normal mouse embryos. This data was previously analyzed [8] to identify a putative repressive transcription factor, nlz2 (zinc finger protein 503), which, when its expression was blocked in zebrafish embryos, led to incomplete optic fissure closure, a coloboma model. The interaction of transcription factors, binding sites and gene networks involving nlz2 and related genes, however, are poorly understood [8]. The present paper is dedicated to develop a novel pipeline for the analysis of microarray gene expression data that complements standard approaches and provides a list of candidate genes guiding further experimental analysis of genetic variations.

By developing and applying a novel clustering scheme, we have identified a 50 per cent larger gene cluster (in comparison to PCA and previous hierarchical cluster analyses [8]), whose spatio-temporal gene expressions correlate with nlz2. According to GoMiner, a computational high-throughput tool for biological interpretation of genomic, transcriptomic, and proteomic data, that identifies the biological processes, functions and components of gene clusters [9,10], this larger cluster still shows gene enrichment for its specific functions in the context of Gene Ontology.

Next, using GoMiner, we sought to identify those gene clusters whose co-expressions correlate with processes in eye development. First, we apply a novel clustering scheme that builds on the intertwining of Laplacian Eigenmaps, a nonlinear geometrical data transformation, with *k*-means and hierarchical clustering. To validate the findings, we also use two standard clustering schemes, basic *k*-means and principal component analysis combined with *k*-means and hierarchical clustering. All three methods identify gene clusters enriched for functional GoMiner categories related to eye development, but the proposed nonlinear scheme leads to lower false discovery rates. Secondly, we have proposed a mechanism that allows experts to introduce their input in form of additional, labeled information by means of a potential on a data-dependent graph in [11] to improve the dimension reduction and clustering process. Distances between certain labeled genes are forced to appear closer than normally while others are increased. In the present paper, we aim to label genes that are highly connected and thus constitute hubs within the regulatory network. Such genes appear to promote coherence within a gene cluster and would thus be ideal candidates for labeling to obtain a more meaningful and coherent clustering. There are many ways to extract genes of high connectivity, and we use the weights that are generated by the Laplacian on the regulatory network and alternatively weighted correlation networks as described in [12]. Identified gene hubs are then labeled to incorporate regulatory network characteristics into the labeled Laplacian clustering. This novel clustering scheme based on nonlinear dimension reduction and involving labeled data further improves the biological specificity according to GoMiner analysis.

Starting from experimental work based on LCM and microarray technologies in organogenesis, we obtain a list of candidate genes that could be significant in normal development of optic fissure closure and could be useful in guiding analysis of genetic variations in humans with coloboma.

## Materials and methods

The Affymetrix MOE 430 2.0 microarray datasets analyzed to develop and test our new method were for eight samples LCM microdissected from serial cryosections of the retina at the site of final optic fissure closure in the mouse embryos at specific embryonic stages 10.5 days through 12.5 days previously reported in [8]. The 8 time-points span the time just before and just after final fusion (optic fissure closure) and were expected to reveal sets of genes critical for the completion of optic fissure closure in normal development. This previous report further investigated a specific putative repressive transcription factor, nlz2 (or zinc finger protein 503), that was discovered to be highly expressed before and during fissure closure and then downregulated. Gene knockdown experiments in zebra fish of nlz2 resulted in incomplete optic fissure closure (coloboma). Our current analysis explores possible associated gene regulation patterns. Within the 8 different time-point microarrays were 8316 genes consistently identified as expressed and with greater than 2-fold variation in gene expression levels. For our clustering analysis, we chose the subset of *n* = 3416 genes whose expression levels varied between 4-fold and 26-fold over the 2 days of embryonic development. For analysis purposes, each gene of the microarray is considered as a vector of its expression levels. This perspective yields a collection of *D* = 8 dimensional vectors. Our proposed analysis relies on Laplacian Eigenmaps [13,14], a geometrical data transformation that provides a new representation of gene expressions still covering essential geometrical behaviors. The nonlinear geometric representation can be further steered by involving labels [11] that are either derived from weighted correlation networks analysis [12] or from the Laplacian analysis. We intertwine this new data representation with *k*-means [15], a widely used clustering scheme. GoMiner [9,10] is then used to identify genes within clusters that are associated with particular biological processes or function. Let us list the steps of our proposed scheme:

1. **Expression vectors:** Each gene’s expression over the 8 time points builds a vector. They constitute a collection {*x*_1_,…, *x_n_*} of 8-dimensional vectors, where *n* is the number of considered expressions

2. **Nonlinear dimension reduction:** Choose a target dimension *d* <*D*, and obtain a new *d*-dimensional data representation {*y*_1_,…, *y_n_*} of the original *D*-dimensional vectors {*x*_1_,…, *x_n_*}

3. ***k*-means:** Run *k*-means on {*y*_1_,*…*,*y_n_*} to obtain the final clustering

4. **GoMiner:** Feed the clusters into GoMiner to evaluate their biological relevance

Step 2 in the above scheme is specified in two different ways: First, we use a nonlinear dimension reduction method without labeling (unsupervised):

2.A **Laplacian Eigenmaps:** Choose the number *m* of gene neighbors and a target dimension *d* <*D*, then apply Laplacian Eigenmaps to obtain a new *d*-dimensional data representation {*y*_1_,*…*,*y_n_*} of the original *D*-dimensional vectors {*x*_1_,…,*x_n_*}

Alternatively, we may want to incorporate further input into the dimension reduction process by using labeled data. We then identify step 2 with the following supervised procedure:

2.B a) **Identifying highly connected genes:** Apply an R package for weighted correlation network analysis (WGCNA) [12] to identify genes that are highly connected within the gene regulatory network and that act as hubs. Alternatively, use the Laplacian analysis to identify highly connected genes

2.B b) **Schroedinger Eigenmaps:** Gene hubs are labeled by means of a potential term. Choose the number m of gene neighbors and a target dimension *d* <*D.* The application of Laplacian Eigenmaps with potentials [11] yields a new *d*-dimensional data representation {*y*_1_,*…*,*y_n_*} of the original *D*-dimensional vectors {*x*_1_,…,*x_n_*}

In the following, we present the components of the above scheme in more detail. For comparison we also applied PCA and *k*-means and therefore briefly discuss these conventional methods too.

### Principal component analysis

PCA [16] is a statistical tool that linearly transforms the data into an orthogonal coordinate system whose axes correspond to the principal components in the data, i.e., the first principal component accounts for as much variance in the data as possible and, successively, further components capture the remaining variance. Through an eigenanalysis, the principal components are determined as eigenvectors of the dataset’s covariance matrix and the corresponding eigenvalues refer to the variance that is captured within each eigenvector. After subtracting the mean of the dataset, PCA is performed on vectors {*x*_1_,…,*x_n_*} by first diagonalizing the covariance matrix cov(X) = *E*(*XX*^⊤^), where *X* = (*x*_1_…*x_n_*) is the zero mean data matrix. The eigenvectors *p*_1_,*…*,*p_D_ -* the principal components ordered according to the magnitude of their eigenvalues - provide the transformed data *Y* = *W^⊤^ X*, where *W* = (*p*_1_*…p_D_*)*.* We obtain the collection of d-dimensional vectors {*y*_1_,…,*y_n_*} whose first entries represents the abundance of the primary principal. The second entries are each datapoint’s projection along the second eigenvector and so forth.

### Laplacian Eigenmaps

Laplacian Eigenmaps (LE) [13,14] is a nonlinear geometric tool that transforms data into a new representation in a nonlinear fashion. Given points {*x*_1_,…,*x_n_*} ⊂ ℝ*^D^*, we assume that they are steered by *d* latent variables, and aim to find a new data representation {*y*_1_,…, *y_n_*} ⊂ ℝ^*d*^. We briefly recall the three step procedure of Laplacian Eigenmaps.

**Step 1: Adjacency graph, *m*-nearest neighbors** We build a graph *G*, whose nodes *i* and *j* are connected if *x_i_* is among the *m*-nearest neighbors of *x_j_* or vice versa. The distance between data points is measured by the Euclidean metric. The graph *G* represents the connectivity of the data vectors.

**Step 2: Heat kernel as weights** Next, we weight the edges of the graph and focus on the diffusion *weight matrix W* given by(1)

The number of neighbors *m* controls the sparsity of *W.*

**Step3: Solving an eigenvalue problem** We denote a potential new data representation by *y* = (*y*_1_,…,*y_n_*)^⊤^, where each row is considered as a vector in ℝ*^d^*, and we then consider the following minimization problem(2)

where *L* = *D — W* and *D* is the diagonal matrix *D_i,i_* = ∑*_j_W_i,j_*. The minimizer of (2) is given by the *d* minimal eigenvalue solutions of *Lx* = *λDx* under the constraint *y*^⊤^*Dy* = *I*, where *I* denotes the identity matrix, i.e., the minimizer *y*’s columns are the *d* eigenvectors with respect to the smallest eigenvalues. If the graph is connected, then **1** = (1,…,1)^⊤^ is the only eigenvector with eigenvalue 0, and we exclude it. Instead of (2), we try to find the minimizer of(3)

By applying the change of variables *z* = *D*^1/2^*y*, this yields(4)

where . The minimizer *z* is given by the *d* eigenvectors with smallest nonzero eigenvalue, and we obtain the *d*-dimensional representation {*y*_1_,…,*y_n_*} from *y* = *D^-1/2^ z.*

### Identifying highly connected genes

Weighted gene co-expression network analysis is a systems biology tool that allows to identify highly connected genes within a regulatory network. An R package implementation WGCNA is available with an accompanying tutorial [12]. Alternatively, the matrix *D* in (2) is a measure of the connectivity within the network and can be used to identify highly connected genes within the Laplacian framework directly.

### Schroedinger Eigenmaps

Based on the Laplacian matrix *L* in (3), a flexible potential, that can capture additional labels, has been introduced in [11]. The matrix *L* is replaced with a Schroedinger type matrix *E* = *L* + *V*, where *V* is a potential matrix that encodes labels. One then aims to minimize(5)

The result is a new Schroedinger Eigenmaps method that allows for input in an otherwise fully automated dimension reduction process [11]. Here, labels are utilized to emphasize “important” genes, and we use the connectivity of genes as a measure of their importance in the description of the regulatory network.

### Standard cluster analysis

For hierarchical clustering, we refer to [17], and we also apply a shape similarity-based clustering as introduced in [18]. *k*-means is a method of cluster analysis which aims to partition *n* observations into *k* clusters {*c*_1_,…,*c_k_*}, where *k* has to be chosen a-priori [15], i.e., one aims at minimizing

where  is the mean of cluster *c_j_*. The basic *k*-means algorithm requires the target number of clusters to be specified as a parameter.

The *k*-means algorithm begins with a data set, a target number of clusters *k*, and a set of *s*_1_,…,*s_k_* initial cluster centroids. It then iteratively assigns points to clusters by centroid proximity, and then adjusts centroids to reflect changes in cluster membership. The algorithm terminates either after a specified number of iterations, or once the cluster centroids/membership no longer change. Although optimal results cannot be guaranteed, the algorithm is quite fast, and many runs can be efficiently computed, with the best clustering taken as an overall result.

### GoMiner

GoMiner provides a quantitative and statistical analysis-tool for biological interpretation of genomic, transcriptomic, and proteomic data, commonly derived from gene expression microarray experiments. It classifies genes into biologically coherent categories and then uses the Gene Ontology project to identify the biological processes, functions and components of genes within these categories [9,10]. A one-sided Fisher’s *p*-value is used to determine the significance and biological enrichment levels within a category.

### Clustering with Genesis

Clustered image maps (CIMs) were first introduced in [19] and are produced here with the Genesis program [20]. We select the Euclidean distance metric and average linkage for hierarchal clustering. To facilitate visualization, a recently-added feature of GoMiner has been implemented that removes large generic categories from all CIMs.

### Silhouette coefficient

The silhouette coefficient is a measure for the coherence of clusters. If we take a clustering *C* to be a mapping from a data set *X* = {*x*_1_,…,*x_n_*} to the integers 1, 2, …, *k* (where *k* is the total number of clusters), we can define the silhouette coefficient *sil*(*x*) for each point *x* in *X* to be

where *A*(*x*) is the average distance between *x* and other points in its cluster, and *B*(*x*) is the average distance between *x* and the points in the nearest neighboring cluster, cf. [21]. The silhouette coefficient *sil(i)* for a cluster *i* is the average of the coefficients for its constituent points. We similarly define the silhouette coefficient sil for an entire clustering to the average silhouette coefficient over all data set points. A clustering with a silhouette coefficient closer to 1 will contain more cohesive and well-separated clusters. For our experiments, we use the squared Euclidean distance for the computations indicated above, as well as for the data clustering algorithms.

### Description of the approach

**Figure 1:**
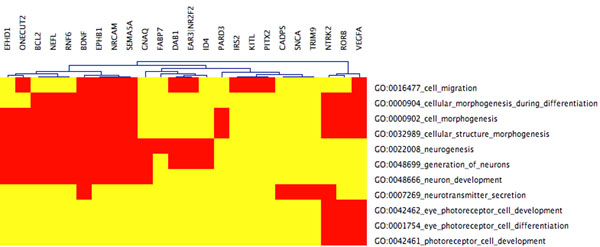
**CIM cluster 22** CIM for LE+k-means cluster 22 with functional categories related to eye development, false discovery rate (FDR)< 0.05. The cluster is enriched for eye photoreceptor cell development and for a eye photoreceptor cell differentiation. We hence see GO categories that are closely related to eye development although the FDR is stringently chosen. 24 genes are mapped to 11 GO functions. (Red: genes are mapped to GO categories, Yellow: no association)

**Figure 2:**
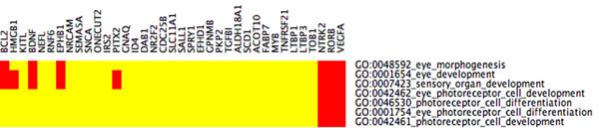
**CIM cluster 22 with relaxed** FDR portion CIM for LE+k-means cluster 22 with functional categories related to eye development (the entire CIM contains 74 GO categories). The input cluster for the present CIM is the same as for Figure 1. By choosing the less stringent FDR< 0.15, more GO categories are statistically enriched, and 36 genes (only 24 in Figure 1) are mapped to these GO categories. Beside the eye related categories in Figure 1, there are additionally eye morphogenesis, eye development, and sensory organ development.

**Figure 3:**
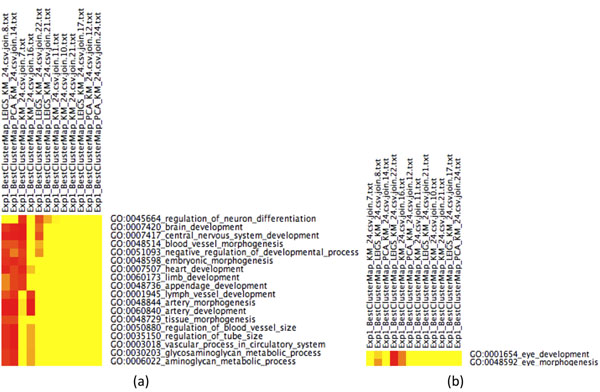
**CIMs across methods** Enriched GoMiner categories that are shared among clustering methods. Each of the 3 different clustering methods (LEIGS KM 24, PCA KM 24, KM 24) produced 24 clusters. We picked 12 among the 72 clusters that seemed to show significant enrichment by means of GO categories. (Yellow means no association. The darker red, the stronger the association between cluster and category) Cluster 22 from Laplacian Eigenmaps+*k*-means has shown eye related GO categories in Figures 1 and 2 with very stringent FDR. Figures 3 a)-b) verify that these categories have not been picked by chance and that the proposed Laplacian-based scheme leads to lower false discovery rates than the other methods and hence appears to provide greater biological specificity and sensitivity. (a) GO categories that are shared by at least three clusters, FDR< 0.10. First, cluster 8 of Laplacian Eigenmaps+*k*-means appears to be closely related to cluster 14 derived from PCA+k-means. Cluster 22 from Laplacian Eigenmaps+k-means shares few biological functions with cluster 7 of *k*-means. However, GO categories that are related to eye development are not shared by any other method at FDR< 0.10. Recall that cluster 22 from Laplacian Eigenmaps+k-means has shown enrichment for these categories already at FDR< 0.05 in Figure 1. (b) The portion CIM with FDR< 0.20 that is associated to additional eye development categories that weren’t present in Figure 3a). They are shared by the Laplacian+*k*-means cluster 22, by *k*-means cluster 16, and by Laplacian+*k*-means cluster 8. The entire CIM contains too many GO categories to be listed here.

**Figure 4:**
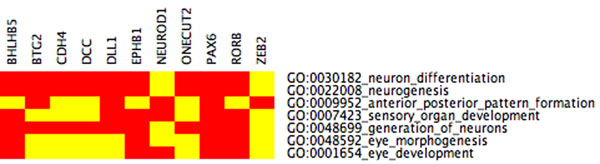
**CIM Schroedinger Eigenmaps I** Seven highly connected genes from Figures 11 and 12 were labeled in Schroedinger Eigenmaps. After clustering, all seven labeled genes are contained in the same cluster with 145 other genes. The cluster is enriched for categories (eye morphogenesis, eye development) that are more specific to eye development than the results without labeling suggesting that data-dependent gene labeling can increase the biological specificity.

**Figure 5:**
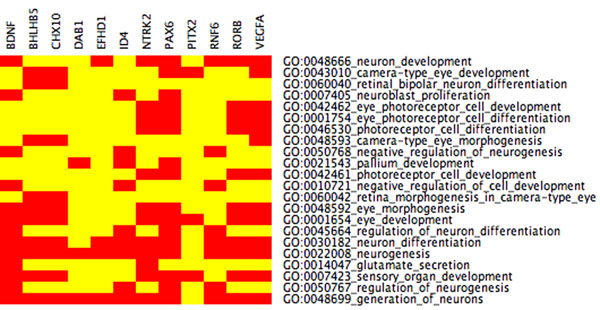
**CIM Schroedinger Eigenmaps II** Five highly connected genes from Figure 13 were labeled in Schroedinger Eigenmaps. After clustering, all five genes are contained in the same cluster. The GO categories for this cluster match the optic fissure closure and thus provide distinct biological specificity (eye photoreceptor cell development, eye photoreceptor cell differentiation, camera type eye morphogenesis, retina morphogenesis in camera type eye, eye morphogenesis, eye development, sensory organ development). Schroedinger Eigenmaps using labels derived from the Laplacian weight matrix D provide better specificity than using Laplacian Eigenmaps without any labels.

Microarray data from LCM isolated cells in a mouse model of coloboma as described in the present Section are analyzed by using standard cluster analysis and a novel gene clustering scheme. We derive a coherent clustering and make use of GoMiner to identify those genes identified in public databases as being associated with eye development or function as a measure of the quality of the other members in the cluster. We used GoMiner to identify the degree of association of clusters obtained by all methods with early stage retinal development, and, in particular, with the closure of the optic fissure, see Figures 1, 2, 3, 4, 5. For *k*-means, we set the target number of clusters to be 24, based on previous work with the current data set [8] that yielded biologically meaningful (but smaller and fewer) cluster results. The maximal silhouette coefficient sil specifies the best *k*-means clustering over 100 repeated runs, starting in each case from different randomly selected initial centroids. The maximum was stable over different 100 run sets, suggesting that an at least near optimal clustering was being obtained. Since the parameter space is too big for an exhaustive search in the dimension reduction process, we fix σ = 1/8 in (1) and assess remaining parameters (number of nearest neighbors and target dimension) over *>m* = 5, …, 10, 12, 15, 20, 25, 50, 100 and *d* = 1, …, 10, 12, 16. The idea is that parameter combinations that yield better cluster structure in the mapped data {*y*_1_,…,*y_n_*} might be better tuned to resolve possible intrinsic structure in the original data {*x*_1_…,*x_n_*}. Silhouette coefficients suggest values *m* = 10 and *d* = 2, which additionally provide excellent GoMiner gene identifications.

## Results

We aim to increase our understanding of the gene network underlying the closure of the optic fissure during vertebrate eye development:

### Enlarged cluster containing nlz2

**Figure 6:**
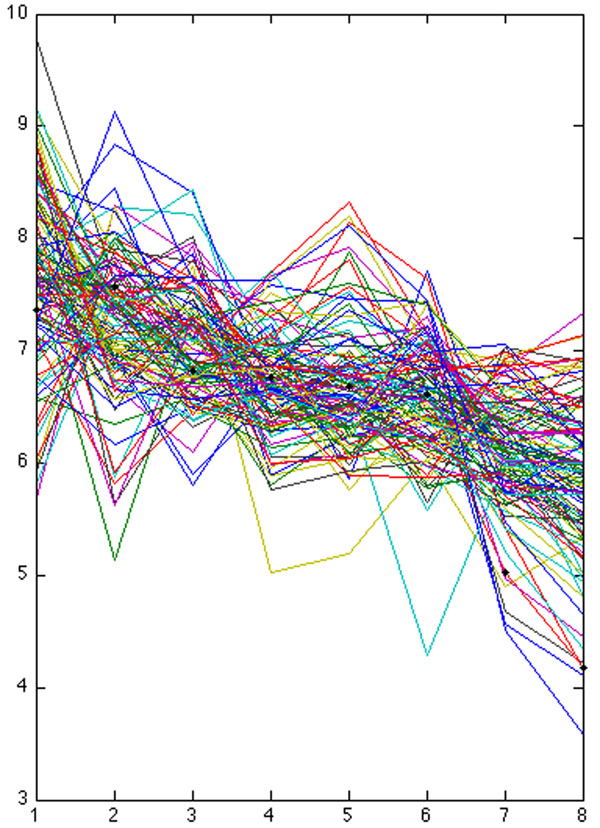
**nlz2 cluster profile** Profile of cluster that contains nlz2. Gene expression levels are plotted vs. 8 time-points, black circles indicate nlz2.

**Figure 7:**
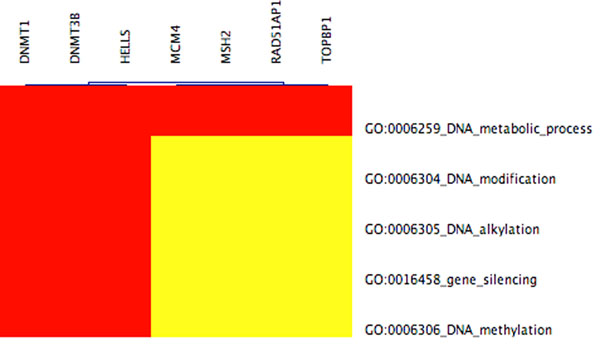
**CIM containing nlz2** Clustered Image Map (produced by GoMiner) showing enriched functional categories for the cluster that contains nlz2 and 168 other genes. More genes in this cluster have been associated to the 5 above GO categories (gene silencing is among them) than one would expect by chance. The 7 genes (DNMT1,…,TOPBP1) above are mapped to these GO categories within the GoMiner database. Red indicates that genes were mapped to GO categories. Yellow means no annotation. Due to gene expression co-variation within the cluster, other genes in the cluster could possibly related to the above GO categories too. Since gene silencing is associated to this cluster, one may speculate that nlz2 and co-varying genes have repressive function and that there is a cluster that shows the reverse expression profile, see Figure 8.

**Figure 8:**
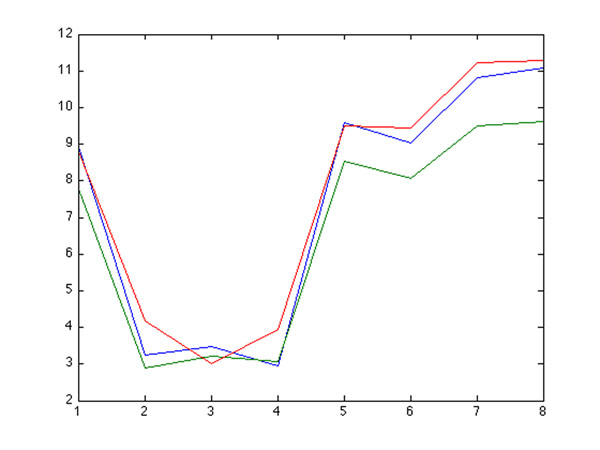
**outliers** Outliers that LE+*k*-means captures into a separate cluster, the associated Affymetrix probes are 1427262_at, 1427263_at, 1436936_s_at. All three probes are associated to XIST, a gene that is transcribed and spliced but does not appear to encode a protein. XIST inactivation is known to be an early developmental process in mammalian females.

We have identified a 50 per cent larger gene cluster than with hierarchical clustering in [8] whose spatio-temporal gene expressions significantly correlate with nlz2, a gene which when previously inhibited in zebrafish induced coloboma. The latter cluster is associated with 210 Affymetrix probes corresponding to 169 genes, nlz2 was among them. See Figures 6 and 7 for gene expression profiles and its set of enriched functional categories. GoMiner assigns the functional category of ‘gene silencing’, indicating the repressive influence of nlz2 and co-varying genes. Previous biological studies have shown nlz2 gene product to repress gene transcription of a number of genes regulated hindbrain development possibly as part of a transcription factor complex consistent with its H2N2 zinc finger domain and its binding site for histone deacetylase. Consistent with this hypothesis, we also identify an additional cluster that varies inversely with the primary ‘nlz2 cluster’ gene silencing, suggestive of the previously documented role of nlz2 in suppression of gene transcription, cf. Figure 8.

### One complementary cluster

We have found a large cluster whose shape is distinct from nlz2 by applying the similarity-based shape clustering in [18]. GoMiner assigns a number of significantly associated functions to this large cluster including **retina morphogenesis** (vertebrate eye), **generation of neurons**, cellular morphogenesis during differentiation, photoreceptor differentiation, cell motility, neuron differentiation, cell projection organization, and biogenesis. The highlighted functions are specifically associated with CHX10, a gene in this cluster that has previously been identified in retinal development, see, for instance, [22,23].

### Collection of enriched clusters

We also apply *k*-means on the original data set and on PCA and LE reduced data. The selected ‘best’ *k*-means result applied directly to the original data has an overall silhouette coefficient of 0.38. To evaluate PCA+*k*-means, for each possible number of retained principal components, the mapped data is clustered, and overall silhouette scores are obtained. The best results refer to the mapping based on principal components capturing about 85% of the variance, with the best overall silhouette score being 0.698. The silhouette scores in the mapped data are substantially higher than those obtained following clustering of the original data, illustrating the fact that Laplacian Eigenmaps enhance cluster structure, see Table 1 for more details.

**Table 1:** comparison for unsupervised methods: Silhouette coefficients and number of genes for each cluster and unsupervised clustering method (no labels). Laplacian Eigenmaps+*k*-means leads to higher silhouette coefficients.

	*k*-means		PCA+*k*-means		LE+*k*-means	
cluster	sil	# genes	sil	# genes	sil	# genes
1	0.0200	65	0.7329	126	0.6535	103
2	0.3067	146	0.6221	60	0.7049	125
3	0.4078	180	0.7002	168	0.6862	174
4	0.4068	234	0.6840	198	0.6848	154
5	0.3401	255	0.7423	157	0.7831	97
6	0.2960	252	0.7033	130	0.7949	389
7	0.3442	90	0.6795	126	0.7369	120
8	0.6509	9	0.6800	65	0.6953	270
9	0.3900	254	0.6393	190	0.7800	91
10	0.2162	34	0.7130	187	0.7046	79
11	0.3056	112	0.6517	182	0.7606	141
12	0.3531	165	0.7162	155	0.7487	122
13	0.4636	182	0.6925	117	0.9889	3
14	0.4267	167	0.7422	205	0.7118	125
15	0.6529	114	0.6968	184	0.5997	85
16	0.1593	86	0.5266	9	0.7214	236
17	0.5488	13	0.6792	84	0.6839	83
18	0.4323	253	0.6956	211	0.7380	135
19	0.1749	20	0.7151	118	0.6466	72
20	0.3076	133	0.6926	170	0.7243	121
21	0.4314	174	0.7041	115	0.7461	199
22	0.4394	130	0.7342	116	0.7442	275
23	0.4538	210	0.7252	192	0.6849	115
24	0.4366	138	0.6792	151	0.8534	102

We find that PCA+*k*-means, basic *k*-means, and LE+*k*-means yield several significantly enriched gene clusters (out of a total of 24) associated with developmental processes, cf. Table 2. Cluster 22 of the Laplacian Eigenmaps-based approach reveals a cluster significantly enriched (with a false discovery rate (FDR) of less than 0.05) for genes specifically implicated in eye development - which is the focus of the experimental work underlying the data set considered in this study. These functional categories (in GoMiner terminology) are

**Table 2:** The number of enriched Go-categories are counted over all 24 clusters at a false discovery rate of 0.05 which is the default configuration of GoMiner. *k*-means and PCA+*k*-means do not show any eye specific enrichement in any of the clusters. Only LE+*k*-means provides one cluster that is enriched for 3 categories specific to eye development. These categories would have even been picked at an FDR of 0.01 suggesting strong statistical support for the LE+*k*-means performance. Potential nonlinear structures in the data could be an explanation for this observation, see Figure 13.

	*k*-means	PCA+*k*-means	LE+*k*-means
# enriched Go-categories	55	17	27
# enriched Go-categories specific to eye development	0	0	3

(i) GO:0042462 eye photoreceptor cell development,

(ii) GO:0001754 eye photoreceptor cell differentiation,

(iii) GO:0042461 photoreceptor cell development.

**Figure 9:**
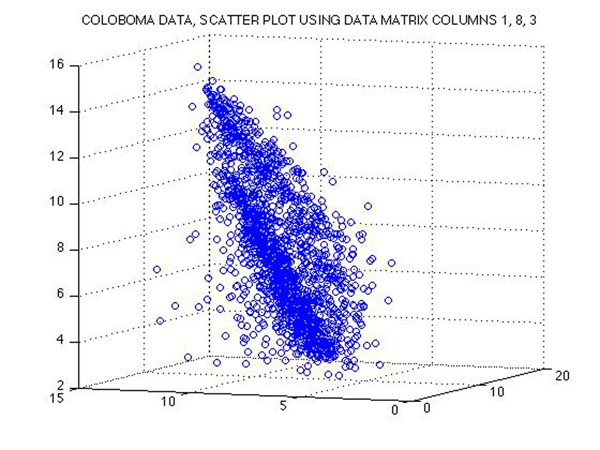
**The 8-dimensional data are projected onto a 3-dimensional subspace** The 3-dimensional subspace is spanned by their 1st, 3rd, and 8th coordinates. If the data would lie on a linear subspace in ℝ^8^, then the projected data must show a linear pattern. However, the actual projection of our data does not show a linear pattern but rather two cones next to each other. A nonlinear approach like Laplacian Eigenmaps could be useful to recover nonlinear structure of the data manifold.

When slightly relaxing the FDR up to < 0.15, this cluster 22 shows gene enrichment for further eye specific developmental functions:

(iv) GO:0048592 eye morphogenesis,

(v) GO:0001654 eye development,

(vi) GO:0046530 photoreceptor cell differentiation,

see also Figures 1 and 4. These categories are neither hit by *k*-means nor PCA+*k*-means clustering when restricting the FDR to < 0.05. By relaxing the FDR, however, both k-means and PCA+k-means clustering show gene enrichment for eye specific functions. This verifies that the eye specific functions in LE+*k*-means cluster 22 are real and have not been picked up by chance. To support the latter claim, we compare the enriched categories in the LE+*k*-means cluster 22 with the clusters of the other two clustering methods with relaxed FDR. It turns out that specific eye development functions are present in all three clustering methods, but our proposed Laplacian-based scheme leads to lower false discovery rates, see also Table 2. Potential nonlinear structures in the data could be an explanation for this observation, see Figure 9. A nonlinear dimension reduction method would clearly be better suited to fit nonlinear structures than linear methods. CIMs in Figures 3 a)-b) indicate which clusters across the three methods share common GoMiner categories. It enables us to identify categories that are more specific to one method than to the others. Based on Table 1 the fraction of genes, that are associated to biological functions, are computable for each cluster, method, and false discovery rate.

### Note on LE+*k*-means

We note that relatively unusual expression patterns are often mapped to distinct, outlying clusters by the Laplacian Eigenmaps approach. For example, the three expression patterns indicated in Figure 8 form a distinct cluster under the Laplacian Eigenmaps data representation. They are not as well separated in the original and PCA-mapped data, and are consequently misplaced in inappropriate clusters. This could be a technical explanation for greater biological specificity of Laplacian Eigenmaps clustering.

### Schroedinger Eigenmaps

**Figure 10:**
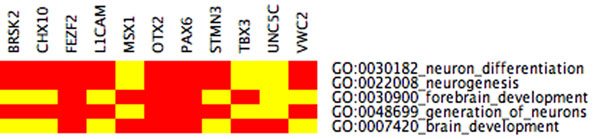
**CIM Schroedinger Eigenmaps III** We have labeled transcription factors (CHX10, OTX2, PAX6) that are known from the literature to be associated to development. We intend to derive a cluster whose GO categories are specific to eye development when starting with labeled genes. Schroedinger Eigenmaps using these labels is applied and the new data representation is then clustered. The three labeled TF are contained in the same cluster with 154 other genes. GoMiner enrichment analysis leads to relatively generic categories that one would expect from the choice of the labeled TF. However, specific eye development categories were not found. This observation suggests that gene labeling based on literature search leads to relatively poor GoMiner enrichments.

**Figure 11:**
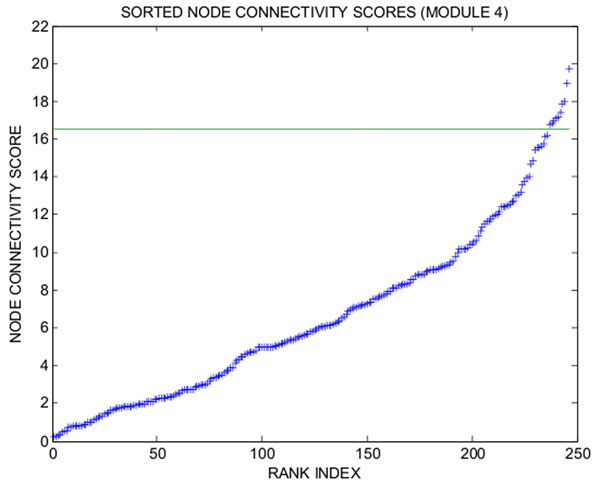
**plot of connectivity scores in increasing order for WGCNA weights** To derive gene labels directly from the measured Affymetrix data rather than from the literature, we aim to identify co-varying genes with high connectivity in the regulatory network. The plot shows the connectivity of Affymetrix probes within a cluster enriched for eye development computed by WGCNA. Rank index refers to Affymetrix probes. The associated most highly connected genes according to WGCNA are Cdh4, Dll1, Tox, Onecut2, Dcc, Epha5, Cadps.

**Figure 12:**
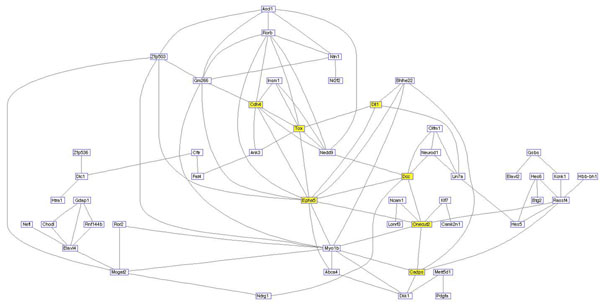
**connectivity network for WGCNA weights** Portion of the thresholded weighted correlation network derived from WGCNA. In the entire connectivity network, each of the genes to be labeled (Cdh4, Dll1, Tox, Onecut2, Dcc, Epha5, Cadps) would have more than 16 connections, see also Figure 11.

**Figure 13:**
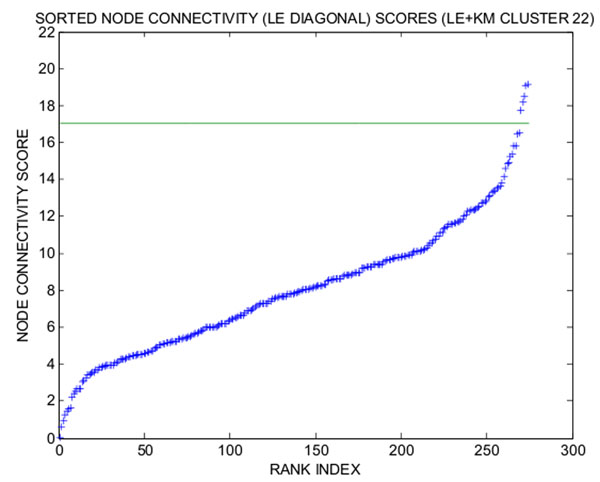
**plot of connectivity scores in increasing order for LE weights, LE cluster 22** We aim to further improve the biological specificity of cluster 22 derived from Laplacian Eigenmaps + *k*-means. To identify co-varying genes with high connectivity in the regulatory network of cluster 22, we measure connectivity by means of the weight matrix D in (2). The connectivity of Affymetrix probes within LE+KM cluster 22 are shown. The most highly connected genes in cluster 22 are Etv3, Zfp386, Kdm4c, Eea1, Fyttd1, which can be used as labels in Schroedinger Eigenmaps.

We first label a collection of transcription factors that are known to be annotated to eye development. Enriched GO categories, however, appear generic when applying Schroedinger Eigenmaps with such labels, cf. Figure 10. To obtain more meaningful labels, that are directly extracted from the data rather than from the literature, we identify a set of highly connected genes through the weighted correlation analysis described in [12], see Figures 11 and 12. These “hub genes” are then labeled by means of the potential to steer Schroedinger Eigenmaps utilizing the gene network topology. This labeling seems to further improve the biological specificity, cf. Figure 4. Alternatively, the matrix D in (2) is a natural measure of the connectivity within the Laplacian framework. According to D, we use highly connected genes as labels within the LE cluster 22, cf. Figure 13, providing the highest biological specificity, cf. Figure 5. Enriched GO categories that are derived from supervised and unsupervised dimension reduction are shown in Table 3. The supervised procedure Schroedinger Eigenmaps identifies more categories specific to early retinal development and the optic fissure closure than the unsupervised approach.

**Table 3:** Comparison between supervised and unsupervised methods: GO categories related to the optic fissure closure that are associated to clusters derived from unsupervised (no data labels) and supervised (labeled data) methods. Using labels that are computed directly from the measured data appears to provide more biological meaningful associations than unsupervised methods.

Unsupervised methods	Schroedinger Eigenmaps + WGCNA / D-labels
eye morphogenesis	
eye development	
eye photoreceptor cell development	
eye photoreceptor cell differentiation	
photoreceptor cell development	
embryonic morphogenesis	
morphogenesis of a branching structure	
sensory organ development	
	camera type eye morphogenesis
	retina morphogenesis in camera type eye
	retinal bipolar neuron differentiation

## Discussion

Obtaining a clearer understanding of the gene regulatory network underlying optic fissure closure during eye development will be a long process involving genetic analysis of humans with coloboma and studies of eye development in animal models. Our present analysis and results focus on expanding a list of candidate genes that could be critical for normal fissure closure and in coloboma patients may contain mutations. Compared with conventional clustering algorithms that we tested, our new method is able to identify larger clusters associated either with the nlz2 gene expression or with a distinctly complementary pattern enriched with associations to eye development gene ontologies. It also uniquely identifies the ‘nlz2-repressed’ pattern as a distinct cluster, cf. Figure 2. The large temporally covarying gene cluster in Figure 7 is identified by GoMiner as being significantly associated with gene silencing, suggestive of a gene regulatory network that represses alternative fates until optic fissure closure is successfully completed (day 11.5 in the mouse). The pattern of genes in Figure 2 could represent such genes that are transiently repressed only when the nlz2 cluster is high. Using temporal pattern-based similarity clustering [18] allows identification of other distinct clusters (i.e., not containing nlz2) in which GoMiner identifies significant associations with specific developmental functions in databases.

Distinct biological specificity for our data set is obtained when labeling highly connected genes and encoding these labels in the potential term. The GO categories **eye morphogenesis, retina morphogenesis in camera type eye, and camera type eye morphogenes**, for instance, reflect the optic fissure closure and are identified by Schroedinger Eigenmaps suggesting that nonlinear dimension reduction with labeled data can improve the biological specificity in gene cluster analysis, cf. Figure 5 and Table 3.

Clearly, our new mathematical approach to identify new components of gene regulatory networks controlling development is preliminary and would need further validation to claim its usefulness in more generality. We anticipate improvements in our analysis methods based on nonlinear dimension reduction with connectivity analysis and labeled data.

## Conclusions

Microarray data are commonly used for global searches for gene expression changes that might be associated with a perturbation of a cell state or in pathology. In organ development, temporal and spatial patterns accessible through microdissection are associated with reproducible changes in gene expression of even larger numbers of genes. More efficient analysis of microarray data from such microdissected samples could provide improved understanding of cell fate and organogenesis as well as elaboration of gene expression covariance networks. Our nonlinear analysis scheme based on Laplacian Eigenmaps and labeling highly connected genes through a potential appears to offer advantages over standard clustering algorithms in the sense of greater biological specificity and sensitivity. Our results motivate further analysis of nonlinear dimension reduction with labeling within other microarray data sets from LCM dissected tissue or other phenotypically specific cell samples to potentially validate its biological specificity in more generality. Together with LCM-focused gene expression microarray measurements, our proposed analysis could be part of an iterative process to more completely identify additional elements in gene regulatory networks underlying mammalian organogenesis.

## Competing interests

The authors declare that they have no competing interests.

## Authors’ contributions

ME, WC, and RFB made substantial contributions to conception and design of the study. BPB and JB acquired the primary expression data. ME, VNR, and BRZ analyzed the data. ME, VNR, and RFB contributed to the interpretation of the data. ME and RFB wrote the manuscript, and ME, VNR, BRZ, WC, and RFB have been involved in revising it critically.
